# Protocol adherence for continuously titrated interventions in randomized trials: an overview of the current methodology and case study

**DOI:** 10.1186/s12874-017-0388-3

**Published:** 2017-07-17

**Authors:** F. Lauzier, N. K. Adhikari, A. Seely, K. K. Y. Koo, E. P. Belley-Côté, K. E. A. Burns, D. J. Cook, F. D’Aragon, B. Rochwerg, M. E. Kho, S. J. W. Oczkowksi, E. H. Duan, M. O. Meade, A. G. Day, F. Lamontagne, for ACCADEMY (Academy of Critical Care – Development, Evaluation, and Methodology)

**Affiliations:** 1 Department of Medicine, Université Laval, Québec, Québec Canada; 2 Department of Anesthesiology and Critical Care Medicine, Université Laval, Québec, Québec Canada; 3 Centre de recherche du CHU de Québec-Université Laval, Axe Santé des Populations et Pratiques Optimales en Santé, Québec, Québec Canada; 4 Interdepartmental Division of Critical Care Medicine, University of Toronto, Toronto, Ontario Canada; 5 Department of Critical Care Medicine, Sunnybrook Health Sciences Centre, Toronto, Ontario Canada; 6 Thoracic Surgery and Critical Care Medicine, University of Ottawa, Ottawa, Ontario Canada; 7 Ottawa Hospital Research Institute, Ottawa, Ontario Canada; 8 Swedish Medical Group, Seattle, Washington USA; 9 Department of Medicine, Western University, London, Ontario Canada; 10 Departments of Medicine, Clinical Epidemiology & Biostatistics, McMaster University, Hamilton, Ontario Canada; 11 Department of Medicine, Université de Sherbrooke, Sherbrooke, Québec Canada; 12 Li Ka Shing Knowledge Institute, St. Michael’s Hospital, Toronto, Ontario Canada; 13 Department of Anaesthesiology, Université de Sherbrooke, Québec, Canada; 14 Centre de Recherche du Centre Hospitalier Universitaire de Sherbrooke, Sherbrooke, Québec Canada; 15 School of Rehabilitation Science, McMaster University, Hamilton, Ontario Canada; 16 Kingston General Hospital Research Institute, Kingston, Ontario Canada

**Keywords:** Clinical trial, Critical care, Protocol deviation, Shock, Pilot studies

## Abstract

**Background:**

The standard definition for protocol adherence is the proportion of all scheduled doses that are delivered. In clinical research, this definition has several limitations when evaluating protocol adherence in trials that study interventions requiring continuous titration.

**Discussion:**

Building upon a specific case study, we analyzed a recent trial of a continuously titrated intervention to assess the impact of different definitions of protocol deviations on the interpretation of protocol adherence. The OVATION pilot trial was an open-label randomized controlled trial of higher (75–80 mmHg) versus lower (60–65 mmHg) mean arterial pressure (MAP) targets for vasopressor therapy in shock. In this trial, potential protocol deviations were defined as MAP values outside the targeted range for >4 consecutive hours during vasopressor therapy without synchronous and consistent adjustments of vasopressor doses. An adjudication committee reviewed each potential deviation to determine if it was clinically-justified or not. There are four reasons for this contextual measurement and reporting of protocol adherence. First, between-arm separation is a robust measure of adherence to complex protocols. Second, adherence assessed by protocol deviations varies in function of the definition of deviations and the frequency of measurements. Third, distinguishing clinically-justified vs. not clinically-justified protocol deviations acknowledges clinically sensible bedside decision-making and offers a clear terminology before the trial begins. Finally, multiple metrics exist to report protocol deviations, which provides different information but complementary information on protocol adherence.

**Conclusions:**

In trials of interventions requiring continuous titration, metrics used for defining protocol deviations have a considerable impact on the interpretation of protocol adherence. Definitions for protocol deviations should be prespecified and correlated with between-arm separation, if it can be measured.

**Electronic supplementary material:**

The online version of this article (doi:10.1186/s12874-017-0388-3) contains supplementary material, which is available to authorized users.

## Background

In clinical practice, adherence is defined as the extent to which a person’s behaviour corresponds with the recommendations made by a healthcare provider [1, 2]. In a research setting, non-adherence hinders the feasibility of clinical trials because patients who do not receive the intended intervention will not be affected by it [3]. In addition, non-adherence may bias trial results because patients who do not adhere to study protocols may have an inherently different prognosis compared to those who comply with the protocol [4]. Accordingly, variations in how protocol adherence is monitored and reported may bear on the interpretation of clinical trials [5–7].

The standard definition for protocol adherence is the proportion of all scheduled doses that are received [8]. This approach, however, is not applicable to interventions requiring continuous titration where a total number of scheduled doses is irrelevant. With interventions like continuous infusions of vasopressors and insulin, as well as mechanical ventilation, there exist no finite number of scheduled doses. In this article, we propose an approach to measure and report adherence in trials of continuous interventions titrated by bedside teams. This approach, informed by a recent pilot trial, acknowledges clinically-justified deviations and contextualizes protocol adherence into a pragmatic framework.

## Discussion

### Case study – The OVATION pilot trial

The OVATION pilot trial was an open-label randomized controlled trial of higher versus lower mean arterial pressure (MAP) targets for vasopressor therapy in shock [9]. The trial interventions were developed with input from clinicians and, with Research Ethics Board approval at each of 11 participating academic hospitals from Canada and the United States, we randomly assigned critically ill patients with vasodilatory shock to a lower (60–65 mmHg) versus higher (75–80 mmHg) MAP target. The primary feasibility objective of the trial was to achieve a 5 mmHg difference in MAP during vasopressor therapy between study groups. Intensive care unit (ICU) research personnel screened and enrolled eligible patients and recorded MAP and vasopressor doses hourly for the duration of vasopressor therapy. This approach enabled the investigators to measure the time within, above, and below assigned target MAP ranges, and to identify whether adjustments to vasopressor infusions complied with the study protocol. From May 2013 to April 2014, 118 patients participated in the OVATION pilot trial and 117 received vasopressor infusions for 695 patient-days.

In this trial, we defined potential protocol deviations a priori as MAP values outside the targeted range for >4 consecutive hours during vasopressor therapy without synchronous and consistent adjustments of vasopressor doses. As such, out of range MAPs did not constitute protocol deviations when the bedside team titrated vasopressors as required (i.e., increased dose if MAP was below the prescribed range or decreased dose if MAP was too high during the 4-h window). This 4-h time window was proposed by the steering committee and approved by all OVATION trial investigators. Moreover, we adjudicated each potential protocol deviation to determine if these events were clinically justified (Additional file 1). This approach was in sharp contrast to the mere reporting of MAP values at selected time points. Herein, we discuss four advantages of this contextual measurement and reporting of protocol adherence in clinical trials of interventions that require continuous titration.

Reason 1: Between-arm separation is a robust measure of adherence to complex protocols.

While protocol deviations may occur with simple interventions, they are more likely to occur in complex interventions. Thus, interventions requiring frequent or continuous titration present the greatest challenge, since protocol deviations may occur at any moment of the trial, in contrast to once or at most several times per day for most other interventions. When designing the OVATION pilot trial, it was not possible to specify an acceptable number of ‘missed doses’ for continuous vasopressor infusions. Instead, the extent of separation in MAP between arms was chosen as a crucial feasibility criterion to proceed to a larger trial. Accordingly, we prespecified what we considered the minimally acceptable separation based on clinical considerations, recognizing that even smaller differences (e.g. difference in MAP <5 mmHg) may be statistically significant but clinically unimportant. We observed a mean difference of 9 mmHg (95% confidence interval: 7 to 11 mmHg; *p* < 0.01) in average MAP between arms on days with vasopressors administered, thus exceeding the predefined threshold for feasibility of 5 mmHg.

Reason 2: Adherence assessed by protocol deviations varies in function of the definition of deviations and the frequency of measurements.

The ‘80% rule’ refers to the proportion of all scheduled doses that are received and is often cited as the cutoff for acceptable adherence for pharmacological interventions [10]. As mentioned above, with continuous interventions the number of scheduled doses is not finite. As the frequency of monitoring increases, so do the resources required to track protocol adherence. Close monitoring of protocol deviations is unlikely in trials that lack resources, introducing a risk of ascertainment bias. In contrast, with very frequent monitoring of continuous interventions, the likelihood of recording protocol deviations increases but the clinical impact of each deviation diminishes proportionately. Similarly, criteria for protocol deviations that are too sensitive would exaggerate the impact of protocol deviations beyond what is clinically important and unduly undermine the apparent feasibility and internal validity of the trial.

In the OVATION pilot trial, we considered reporting non-adherence as the proportion of hourly MAP values that were out-of-range during vasopressor therapy (Table 1a). This approach was dismissed on the basis that brief fluctuations in MAP values, randomly distributed above and below the targeted range, would not capture meaningful protocol deviations. Instead, we defined potential deviations as MAP values continuously above or below the targeted range for four consecutive hours (Table 1b). Furthermore, acknowledging that clinicians might adhere to the protocol but the patients fail to respond to the intervention, adjustments to vasopressor infusions were incorporated into the definition for protocol deviations. We considered that blood pressure alone was a poor marker of protocol adherence and that compliance with medication delivery was equally important.

**Table 1:** Protocol adherence according to different definitions

		Higher MAP	Lower MAP	*p* value
		(75–80 mmHg)	(60–65 mmHg)	
		*N* = 58	*N* = 59^a^	
a. Proportion of total hours on vasopressors in and out of range^b^				
*Each patient weighted equally*				
Time within range		33% (30–37%)	28% (24–32%)	0.053
Time above-target		39% (34–45%)	66% (61–71%)	<0.001
Time below-target		28% (22–33%)	6% (4–9%)	<0.001
*Patients weighted proportionally to total hours on VP*				
Time within range		32% (29–35%)	31% (28–34%)	0.81
Time above-target		34% (30–38%)	61% (56–65%)	<0.001
Time below-target		34% (30–39%)	8% (3–13%)	<0.001
b. MAP out of range for four consecutive vasopressor hours				
Number of occurrences of four consecutive hours/vasopressor days (mean events per day)		821/378 (2.2)	681/314 (2.2)	No important differences
Number of days with MAP out of range for at least four consecutive hours (%)		299/378 (79%)	259/314 (82%)	0.29
Number of patients with at least one occurrence of MAP out of range for four consecutive hours (%)		56/58 (97%)	57/59 (97%)	1.00
c. MAP values outside the targeted range for four consecutive hours without synchronous and consistent adjustments of vasopressor doses				
Number of deviation events (mean events per day)		32 (0.08)	43 (0.14)	0.03^c^
Number of days with at least one deviation event (%)		31 (8%)	40 (13%)	0.03^d^
Number of patients with at least one deviation event (%)		26 (45%)	20 (34%)	0.26^e^

*MAP* Mean arterial pressure

^a^This excludes 1 patient who did not have a vasopressor infusion

^b^The proportion of total hours on vasopressor and within, above or below target were calculated by first determining the proportion of hours on vasopressors that were within, above or below target for each patient. The patient level data was then used to calculate the target group means, confidence intervals and *p*-values comparing the higher and lower MAP target groups. The group level summary statistics were first calculated weighting each patient equally regardless of hours on vasopressors and were then re-calculated after weighting each patient proportionally to the number of hours they were on vasopressors

^c^ *P*-value calculated by generalized estimating equations (GEE) with exchangeable within subject working correlation, Poison dependent distribution and log-link

^d^ *P*-value calculated by GEE with exchangeable within subject working correlation, Poison distribution, log-link and the log of the number of vasopressors days as offset

^e^ *P*-value calculated Fisher’s exact test

Reason 3: Distinguishing clinically-justified vs. not clinically-justified protocol deviations acknowledges clinically sensible bedside decision-making and offers a clear terminology before the trial begins.

**Fig. 1:**
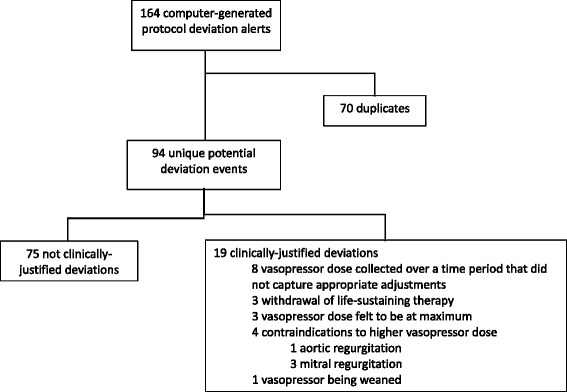
Classification of protocol adherence alerts

The existing literature on protocol adherence focuses mostly on outpatient interventions [2, 11]. In this context, efforts to maximize adherence is directed at study participants. In other settings, such as critical care, protocol deviations do not reflect the behavior of study participants but of the bedside team. During the OVATION pilot trial, a protocol adherence adjudication committee reviewed 164 database-generated protocol adherence alerts, representing 94 unique events. Figure 1 and Table 1c illustrate the classification of protocol adherence alerts. After adjudication, 75 of 94 unique protocol deviation alerts (80%) resulted from situations where the research team could ascertain no justifiable reason for the alert (i.e. these protocol deviations were adjudicated as not clinically-justified). In contrast, 19 protocol adherence alerts corresponded to scenarios where overruling the protocol was “admissible” as per predefined criteria (i.e., clinically-justified protocol deviation). When an unforeseen clinical situation justified the interruption of the trial intervention, we amended the protocol and did not report the event as a protocol deviation, judging these situations to be concordant with usual care [12]. For example, one patient developed acute aortic regurgitation after randomization. This unexpected event required a vasopressor dose reduction and a protocol amendment was prepared for the planned larger trial mandating that clinicians stop the assigned intervention in a similar clinical situation. Such careful assessment of protocol adherence in a pilot study can be used to characterize usual care and provide other useful insights into the acceptability of the intervention. During the OVATION pilot trial, protocol deviations more commonly consisted of MAP values above study targets, even in the higher MAP arm. This was in sharp contrast with stated practices [13]. Thus, by adjudicating protocol deviations, we observed that ICU physicians and nurses appear to be more concerned by the potential risks of hypotension than by the potential risks of vasopressor doses required to achieve higher targets. A contextual analysis of protocol adherence raised the possibility that aggressive vasopressor use has become embedded in standard clinical practice.

Reason 4: Multiple metrics exist to report protocol deviations.

The traditional approach captures the proportion of patients who receive an arbitrary number of scheduled doses. A thoughtful approach is needed to understand the range of reporting options available and to select a metric for reporting protocol deviations that align with the trial objectives. In addition to the criteria that define protocol deviations, the units of measurement also influence the measure of adherence. Choosing to report the number of protocol deviation events, the number of days with at least one deviation event or the number of patients who ever experienced one protocol deviation provides different information. In the OVATION pilot trial, adherence was similar in both arms using the last definition, but was superior in the high MAP arm when we used the first two definitions (Table 1).

### Building on previous work

Various terminologies describe the numerous issues related to protocol adherence. Ellis et al. have categorized non-adherence as ‘erratic adherence’, ‘unwitting non-adherence’ and ‘intelligent non-adherence’ [10]. In some trials, the cessation of a trial intervention prompted by such an event could be considered as a clinically sensible decision with no further comment, a protocol deviation, a protocol violation, or even a serious adverse event without explicit reference to protocol adherence. The distinction between clinically-justified deviations that are not avoidable from the trial perspective and those that are not clinically-justified can be useful to researchers and informed readers of the medical literature [14]. Central and, ideally, blinded adjudication of clinical events and associated protocol decisions can help make these distinctions.

### Strengths and limitations

The strengths of this analysis include a priori definitions for protocol deviations, pilot trial feasibility criteria based on between-arm separation in MAP and total vasopressor dose, as well as parallel comparisons between various metrics. One limitation is that our observations are nested in a single, small clinical trial comparing complex critical care interventions and, thus, do not apply to all settings. Our intention was not to address every methodological issue that relates to protocol adherence. Rather, we used the OVATION pilot trial as a case study to illustrate that failure to contextualize the monitoring, reporting and interpretation of protocol adherence may lead to inappropriate conclusions. Another potential limitation is that there is no objective gold standard to determine the suitability of alternative approaches to the monitoring and reporting of protocol adherence. Admittedly, the definitions of protocol deviations in the OVATION pilot trial were arbitrary (e.g. 4-h time window). However, this is in keeping with every other measure of acceptable adherence, such as the 80% rule, which are typically well accepted. Our intention was not to present a superior definition for protocol adherence. Rather, the purpose of this analysis was to exemplify how trialists who are mindful of the context in which a complex research protocols are delivered may plan the monitoring of protocol adherence. While a clearer taxonomy of issues related to protocol adherence would undoubtedly be valuable to trialists and readers of the medical literature, this project was not designed to achieve this goal. Finally, our work focused on current issues related to protocol adherence. In the future, with the rapid development of real-time, data driven algorithms, the nomenclature for protocol adherence may yet become even more complex.

## Conclusions

Metrics used for defining protocol deviations in a trial evaluating an intervention requiring continuous titration can significantly modify the interpretation of protocol adherence. Protocol deviations should be defined a priori and adapted to the complexity of the clinical context. With more complex interventions, an adjudication process to determine whether the deviations encountered were clinically justified may be warranted.

## Additional file

Additional file 1: Adjudication of potential protocol deviations. (DOCX 18 kb)
