# Supplementary material for: Protocol adherence for continuously titrated interventions in randomized trials: an overview of the current methodology and case study
Source: BMC Med Res Methodol. 2017 Jul 17;17:106. doi: 10.1186/s12874-017-0388-3 (PMC5513343; doi:10.1186/s12874-017-0388-3)
Supplement: Additional file 1: — Adjudication of potential protocol deviations. (DOCX 18 kb) [file 12874_2017_388_MOESM1_ESM.docx]

Additional file 1

*A priori,* we defined protocol deviations as MAP values outside of the targeted range for more than 4 consecutive hours during vasopressor therapy, with no simultaneous attempt to adjust vasopressor dosing. Out of range MAPs did not constitute protocol deviations if vasopressors were titrated as expected (i.e., increased if MAP was below prescribed range or decreased if MAP was too high during the 4-hour window). Throughout the trial, prompt data entry for hourly MAPs and vasopressor doses recorded on electronic case report form (RedCAP^TM^ [1]) allowed real-time identification of potential protocol deviations and rapid feedback to local research teams. The Methods Centre designed an automated audit and feedback tool to provide protocol adherence data to each site. Immediately after the necessary data were entered, this computer algorithm detected potential protocol deviations and sent alerts to the project manager, the principal investigator and the site research team. In addition to these alerts, the data management system generated an electronic protocol deviation form for ICU research staff to collect additional information about the potential deviations. An adjudication committee of four investigators reviewed this information in detail.

The adjudication committee met periodically to review and classify each protocol adherence alert. Predefined scenarios of “admissible” overruling of the protocol arose when the 1) clinical team determined that vasopressor infusions could no longer be increased or decreased safely; 2) patients developed an adverse effect that could plausibly be attributed to vasopressors; or 3) ICU research personnel provided evidence that the hourly recording of vasopressor doses failed to adequately capture dose adjustments. In all other situations, the adjudication committee recorded and coded the reasons for the protocol deviation alert, as reported by clinical caregivers (ICU physicians and nurses, including trainees). In the context of this pilot trial, the research team and the Data Safety and Monitoring Board acknowledged that new knowledge about the acceptability of the protocol might justify future protocol amendments.

References

[1] Harris PA, Taylor R, Thielke R, Payne J, Gonzalez N, Conde JG. Research electronic data capture (REDCap)--a metadata-driven methodology and workflow process for providing translational research informatics support. J Biomed Inform. 2009;42:377-81.
